# Molecular background of Philadelphia chromosome dependent enhancement of cellular growth and tyrosine kinase inhibitor sensitivity

**DOI:** 10.1186/s40164-026-00758-4

**Published:** 2026-02-19

**Authors:** Md Faruq Hossain, Lisa Hagenau, Lars R. Jensen, Johannes Rhode, Thomas Sura, Manuela G. Salazar, Ana Tzvetkova, Corinna Jensen, Stephanie Edwards, Heiko Dunkel, Stefan Simm, Josefine Radke, Andreas W. Kuss

**Affiliations:** 1 Human Molecular Genetics Group, Department of Functional Genomics, Interfaculty Institute for Genetics and Functional Genomics, University Medicine Greifswald, Greifswald, Germany; 2 Department of Functional Genomics, Interfaculty Institute for Genetics and Functional Genomics, University Medicine Greifswald, Greifswald, Germany; 3 Institute of Bioinformatics, University Medicine Greifswald, Greifswald, Germany; 4 Institute for Bioanalytics, Coburg University of Applied Sciences and Arts, Coburg, Germany; 5 Institute for Molecular Genomics, University Medicine Greifswald, Greifswald, Germany

**Keywords:** Philadelphia chromosome, ALL, CRISPR/Cas9, BCR-ABL1 p190, TKI sensitivity, Gene expression, DNA methylation, Proteomics

## Abstract

The Philadelphia chromosome is the result of a balanced reciprocal translocation between the long arms of chromosomes 9 and 22, resulting in the fusion gene BCR-ABL1. Despite it being a hallmark of acute lymphocytic leukemia (ALL), acute myelogenous leukemia (AML) and mixed-phenotype acute leukemia, comparatively little is known about its effects, which can be directly attributed to its presence in cancer cells. To study this question, we created and characterized a Jurkat cell line carrying this alteration via a CRISPR/Cas9-based approach. Compared with wild-type Jurkat cells, BCR-ABL1 p190-expressing cells exhibited increased proliferation and increased sensitivity to tyrosine kinase inhibitors (TKIs). By integrating gene expression, DNA methylation and protein expression data generated by next-generation sequencing (NGS) and mass spectrometry analyses, we identified a number of pathways as well as individual proteins that are altered in association with BCR-ABL1 p190. Among the deregulated proteins, we identified known cancer proteins, such as the tumor suppressors ASS1 and ABI3, which were downregulated in our model, or specifically upregulated TRBC1. Particularly noteworthy is the downregulation of CYP51A1, which is known to confer TKI resistance under normal circumstances, and therefore directly associated with increased TKI sensitivity in BCR-ABL1 p190-positive cells. Another interesting feature is SPART, whose abundance was increased despite strong promoter hypermethylation, indicating that some transcriptional changes in BCR-ABL1 p190-carrying cells occur independently of promoter methylation and reflect broader regulatory effects of the fusion.

**Supplementary Information:**

The online version contains supplementary material available at 10.1186/s40164-026-00758-4.

**To the Editor**

The Philadelphia chromosome (Ph), a chromosomal translocation t(9;22), creates the *BCR-ABL1* fusion gene, a hallmark of certain leukemias, particularly chronic myeloid leukemia (CML) [1]. This fusion leads to a protein that enhances tyrosine kinase enzyme activity, and is supposed to drive uncontrolled cell growth and inhibit programmed cell death via pathways like JAK/STAT and PI3K/AKT [2]. While the p210 *BCR-ABL1* isoform is common in CML, the p190 isoform is frequently found in a subset of acute lymphoblastic leukemia (ALL), particularly in older patients [3]. Detecting fusion transcripts like BCR-ABL1 can support individualized ALL therapy since subcategories of ALL, such as B-ALL, Ph-positive ALL or T-ALL, need different therapies, and their discrimination on the basis of BCR-ABL1 levels allows a higher success rate in treatment selection. Ph-positive B-ALL patients, for example, respond poorly to conventional chemotherapy (e.g [4]). This study aimed to elucidate the molecular effects specifically attributable to BCR-ABL1 p190 expression by introducing the fusion gene into a defined isogenic T-cell background and subsequently analyzing its transcriptional, epigenetic, and proteomic consequences.

Here we present the creation and comprehensive characterization of a Jurkat T-cell leukemia cell line (reviewed previously by ref. [5]) modified to express the p190 BCR-ABL1 fusion protein (Jurkat-Ph), utilizing CRISPR/Cas9 technology (Supplementary material 1). The goal was to establish a model for studying the specific effects of BCR-ABL1 in T-ALL on a defined genomic background. We successfully generated Jurkat-Ph cells by targeting the *BCR* and *ABL1* loci (Fig. 1A). No off-target effects were detected, and cell line identity and genomic stability were verified through short tandem repeat (STR) profiling and whole-genome sequencing (Supplemental Fig. S1). Compared with wild-type (WT) cells, Jurkat-Ph cells exhibited increased proliferation and increased sensitivity to tyrosine kinase inhibitors (TKIs), such as imatinib and dasatinib (Fig. 1B–E). This cell proliferation inhibition was dose dependent in Ph cells but absent in WT cells, highlighting the fusion-specific effect and therapeutic relevance of TKIs [6].

**Fig. 1:**
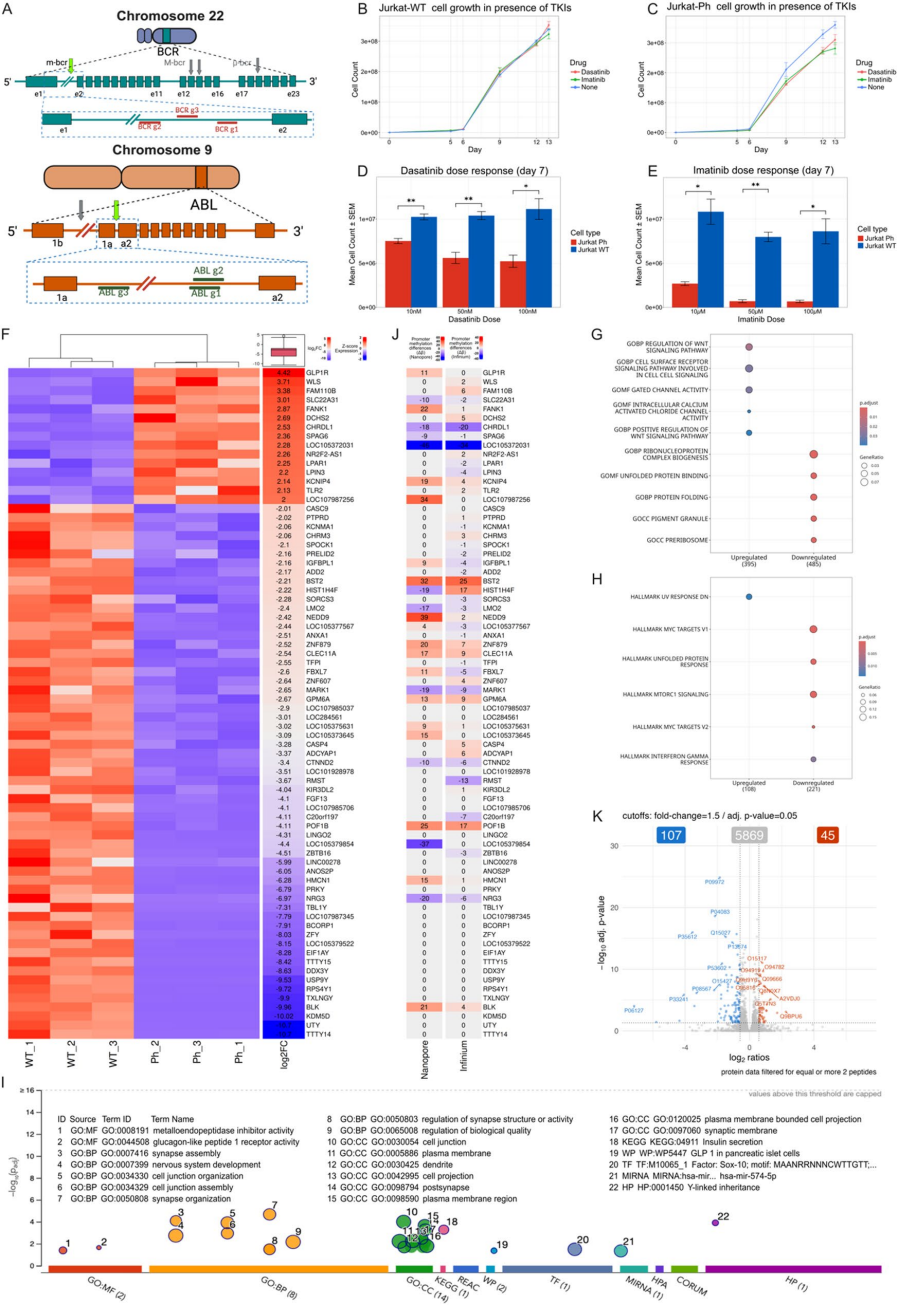
**A** Gene structure and breakpoints of BCR and ABL1. In BCR, most breakpoints in CML occur within the M-BCR region, which encompasses exons 12–15. The m-BCR is located in the 3’ half of the first BCR intron between e1 and e2. The green arrow indicates the region targeted by the sgRNAs. The µ-BCR is located further downstream between exons 19 and 21. In ABL1, the breakpoints are distributed in the intron between exons 1b and 1a or in the intron between exons 1a and 2. The green arrow indicates the region targeted by the sgRNAs (shown in the inset). **B**, **C** Growth curves of Jurkat-WT (B) and Jurkat-Ph (C) cells cultured for 13 days in the presence of *dasatinib* (5 nM) or *imatinib* (50 µM) compared with untreated controls. Cell counts were measured at regular intervals, and the data are presented as the means ± SDs (*n* = 3). Statistical comparisons between Imatinib or Dasatinib and untreated control were performed at each time point; differences did not reach a level of significance (adjusted *p* > 0.05). **D** Dose-dependent effect of imatinib (starting at concentrations of 10, 50, and 100 µM) on Jurkat-WT and Jurkat-Ph cells assessed on day 7. **E** Dose-dependent effect of dasatinib (starting concentrations of 10, 50, or 100 nM) on Jurkat-WT and Jurkat-Ph cells assessed on day 7. All the data are presented as the means ± SEMs from three biological replicates. Pairwise t-tests were performed between Jurkat-WT and Jurkat-Ph cells at each dose; asterisks indicate statistical significance based on adjusted p-values (*p < 0.05, **p < 0.01). **F** Hierarchical clustering heatmap of the top 74 differentially expressed genes (DEGs) in Jurkat-WT and Jurkat-Ph cells (Z scores represent standard normalized expression values). **G** Gene Ontology (GO) over representation analysis (ORA) results of the DEGs. The gene ratio is shown on the x-axis, and the GO terms are indicated on the y-axis. The circle size represents the gene count. The color of the circle represents the adjusted p value. **H** Hallmark ORA. The circle size indicates the number of genes per term, and the color reflects the adjusted p value. **I** Functional enrichment of DEGs in Jurkat-Ph cells. GO term names were manually added to the figure on the basis of the corresponding numerical identifiers. **J** Gene-specific methylation analysis results from Oxford Nanopore direct sequencing (“Nanopore”) and Illumina Infinium MethylationEPIC v2.0 BeadChip array analysis (“Infinium”), comparing the Ph-positive cells with the corresponding wild-type cells. Heatmap values represent differences in promoter methylation levels (Δβ). Positive values indicate promoter hypermethylation whereas negative values indicate promoter hypomethylation, both in Jurkat-Ph cells. **K** Volcano plot showing differentially expressed (log_2_FC ≥ 1.5 and adjusted p value < 0.05) proteins in Jurkat-Ph cells. The upregulated proteins are marked in red, and the downregulated proteins are marked in blue

To investigate the molecular basis of these phenotypic changes, we applied a multiomics approach (Supplementary materials 1). Transcriptomic analysis (RNA-seq) revealed 1168 DEGs (Fig. 1F), associated with pathways involved in WNT signaling, cell surface receptor signaling, and immune regulation (Fig. 1G, H). Specific genes, such as *WLS* (*Wntless*, a gene essential for Wnt ligand secretion and a key regulator of Wnt signaling) and *TLR2* (a Toll-like receptor central to innate immune responses and NF-κB activation) were identified as significantly altered, contributing to leukemic transformation and potentially mediating TKI resistance. Interestingly, *BCR-ABL1* expression was accompanied by a seemingly marked downregulation of Y chromosome genes. However, given that Jurkat cells lack a Y chromosome, this most likely reflects reference alignment artifacts and represents the respective X chromosomal paralogs. Gene set enrichment analysis also indicated enrichment of the insulin secretion pathway. This likely reflects BCR-ABL1-induced alterations in vesicle trafficking, calcium signaling, and exocytosis, which are critical for T-cell activation [7, 8] (Fig. 1I). In parallel, we looked at genome wide DNA methylation, using nanopore sequencing as well as an Illumina Infinium array, which revealed widespread changes in DNA methylation patterns (Fig. 1J).

Proteomic analysis identified 107 downregulated and 45 upregulated proteins in Jurkat-Ph cells (Fig. 1K), with enrichment in pathways related to cell adhesion, immune response, cytoskeleton organization and leukocyte activation (Fig. S2A), indicating impaired immune surveillance and structural integrity. These changes were accompanied by decreased expression of actin-related proteins, which are essential for T-cell mobility and immune synapse formation [9].

Beyond changes in protein abundance, phosphoproteomics analysis showed altered phosphorylation of key proteins, impacting splicing, apoptosis, and proliferative signaling (Fig. 2A). Among them, SRRM1, a splicing factor implicated in AKT pathway activation and oncogenic CD44 isoform switching, was both upregulated and hyperphosphorylated, suggesting a role in leukemic cell proliferation and altered splicing [10, 11].

We next performed an integrated analysis of differentially regulated transcripts and proteins in combination with alterations in genome-wide methylation changes observed in the presence of BCR-ABL1 p190 in order to identify regulatory alterations associated with BCR-ABL1 expression. This analysis revealed 221 genes with coordinated changes (adjusted p value < 0.05) in transcription and promoter methylation, including 13 genes that also exhibited significant differences in protein abundance. The results also showed genes with both canonical (methylation correlated with gene expression) and noncanonical (discordant methylation/expression) regulatory patterns (Fig. 2B-D, Fig. S2B). These mixed regulatory patterns align with recent studies reporting poor genome-wide correlations between promoter methylation and RNA levels (e.g [12]).

**Fig. 2:**
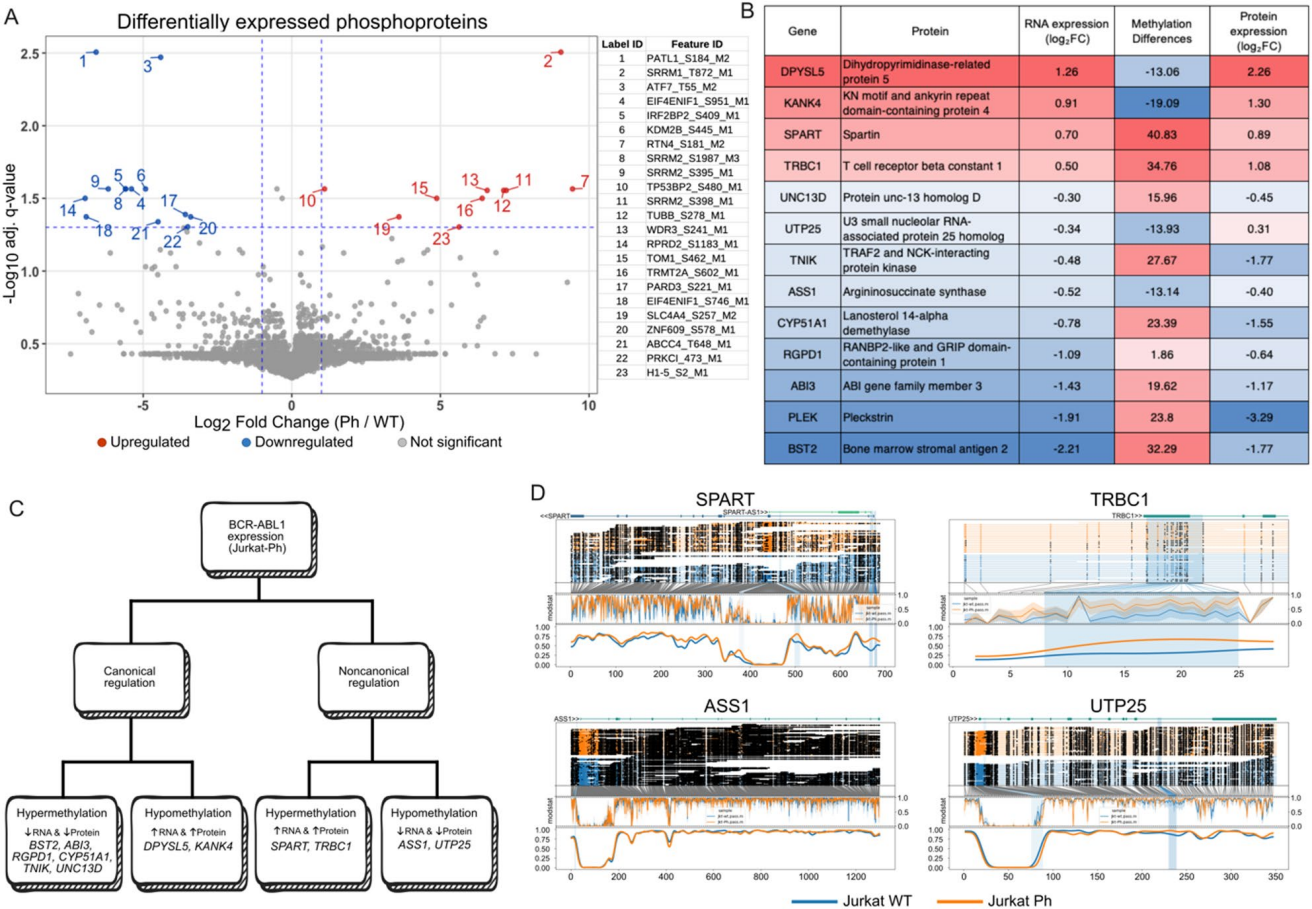
**A** Volcano plot of differentially abundant phosphosites in Jurkat-Ph cells. Phosphorylation sites with an absolute Log₂FC > 1 and false discovery rate (FDR)-adjusted p value < 0.05 are shown, with upregulated sites in red and downregulated sites in blue. The plot highlights significant alterations in phosphorylation patterns associated with BCR-ABL1 expression. **B** Integrated RNA expression, promoter methylation, and protein abundance in Jurkat-Ph cells. **C** Schematic overview of canonical and noncanonical regulatory patterns in BCR-ABL1-positive Jurkat cells. **D** DNA methylation profiles of genes with noncanonical gene regulation in comparison with normally regulated genes. Each panel represents the gene body with promoter and flanking genomic regions, with methylation levels in Jurkat-WT (blue) and Jurkat-Ph (orange) cells. The highlighted boxes indicate differentially methylated regions (DMRs). TRBC1 and SPART are noncanonically upregulated; ASS1 is noncanonically downregulated; and UTP25 has a complex pattern of regulation with a combination of downregulated RNA expression, hypomethylation and upregulated protein expression. The arrow indicates a hypermethylated region in the 5’ area of the SPART-antisense gene SPART-AS1

The Philadelphia chromosome is exceedingly rare in de novo T-ALL, but there are known cases of Philadelphia chromosome–positive T-ALL, including cases with the p190 BCR-ABL1 fusion (e.g [13]). In this context, the Jurkat-Ph model is not intended to reflect disease incidence, but rather to provide a controlled T-cell system to examine the molecular consequences of aberrant tyrosine kinase signaling, which may also be relevant to kinase-driven or Ph-like T-ALL subtype. Taken together our results establish Jurkat-Ph cells as a valuable model for investigating the mechanisms underlying BCR-ABL1-driven T-ALL. In particular, the multiomics data reveal a complex interplay between transcriptional, protein, and epigenetic regulation, providing insights into potential therapeutic targets and the mechanisms of TKI resistance. This dataset will be valuable for future studies aimed at understanding and combating this aggressive leukemia subtype, paving the way for personalized diagnostic and therapeutic approaches for hematologic malignancies. Finally, our findings underscore the importance of considering multiple regulatory layers when investigating the molecular mechanisms underlying cancer development and progression.

## Electronic Supplementary Material

Below is the link to the electronic supplementary material.

Supplementary Material 1.

Supplementary Material 2.

## Data Availability

The datasets generated and/or analyzed during the current study are available in the ProteomeXchange Consortium via the PRIDE repository (accession PXD071195) and in the European Nucleotide Archive (ENA) transcriptomics repository (accession PRJEB104343).

## Abbreviations

ALL: Acute Lymphocytic Leukemia
AML: Acute Myelogenous Leukemia
CML: Chronic Myeloid Leukemia
DEG: Differentially Expressed Gene
DMR: Differentially Methylated Region
FDR: False Discovery Rate
GO: Gene Ontology
ORA: Over Representation Analysis
Ph: Philadelphia Chromosome
STR: Short Tandem Repeat
TKI: Tyrosine Kinase Inhibitors
WT: Wild-Type

## References

[1] Lyu X, et al. A novel BCR-ABL1 fusion gene identified by next-generation sequencing in chronic myeloid leukemia. Mol Cytogenet. 2016. 10.1186/s13039-016-0257-5. 27350795 10.1186/s13039-016-0257-5 PMC4922057

[2] Amarante-Mendes GP, Rana A, Datoguia TS, Hamerschlak N, Brumatti G. BCR-ABL1 tyrosine kinase complex signaling transduction: challenges to overcome resistance in chronic myeloid leukemia. Pharmaceutics. 2022;14(1):215. 10.3390/pharmaceutics14010215. 35057108 10.3390/pharmaceutics14010215 PMC8780254

[3] Gökbuget N. Treatment of older patients with acute lymphoblastic leukemia. Hematol Am Soc Hematol Educ Program. 2016;2016(1):573–9. 10.1182/asheducation-2016.1.573 PMC6142461 27913531

[4] Moorman AV, Chilton L, Wilkinson J, Ensor HM, Bown N, Proctor SJ. A population-based cytogenetic study of adults with acute lymphoblastic leukemia. Blood. 2010;115(2):206–14. 10.1182/blood-2009-07-232124. 19897583 10.1182/blood-2009-07-232124

[5] Gioia L, Siddique A, Head SR, Salomon DR, Su AI. A genome-wide survey of mutations in the Jurkat cell line. BMC Genomics. 2018;19:334. 10.1186/s12864-018-4718-6. 29739316 10.1186/s12864-018-4718-6 PMC5941560

[6] Yuan M, et al. RAG enhances BCR-ABL1‐positive leukemic cell growth through its endonuclease activity in vitro and in vivo. Cancer Sci. 2021;112:2679–91. 10.1111/cas.14939. 33949040 10.1111/cas.14939 PMC8253288

[7] Trebak M, Kinet J-P. Calcium signalling in T cells. Nat Rev Immunol. 2019;19(3):154–69. 10.1038/s41577-018-0110-7. 30622345 10.1038/s41577-018-0110-7 PMC6788797

[8] Lou J, Rossy J, Deng Q, Pageon SV, Gaus K. New insights into how trafficking regulates T cell receptor signaling. Front Cell Dev Biol. 2016;4:77. 10.3389/fcell.2016.00077. 27508206 10.3389/fcell.2016.00077 PMC4960267

[9] Morley SC. The actin-bundling protein L-plastin supports T-cell motility and activation. Immunol Rev. 2013;256(1):48–62. 10.1111/imr.12102. 24117812 10.1111/imr.12102 PMC3801223

[10] Jiménez-Vacas JM, et al. Dysregulation of the splicing machinery is directly associated to aggressiveness of prostate cancer. EBioMedicine. 2020;51:102547. 10.1016/j.ebiom.2019.11.008. 31902674 10.1016/j.ebiom.2019.11.008 PMC7000340

[11] Cheng C, Sharp PA. Regulation of CD44 alternative splicing by SRm160 and its potential role in tumor cell invasion. Mol Cell Biol. 2006;26(1):362–70. 10.1128/MCB.26.1.362-370.2006. 16354706 10.1128/MCB.26.1.362-370.2006 PMC1317625

[12] Spainhour JC, Lim HS, Yi SV, Qiu P. Correlation patterns between DNA methylation and gene expression in the cancer genome atlas. Cancer Inf. 2019;18:1176935119828776. 10.1177/1176935119828776. 10.1177/1176935119828776 PMC6376553 30792573

[13] Kohla S, Kourashy SEL, Nawaz Z, Youssef R, Al-Sabbagh A, Ibrahim FA. P190BCR-ABL1 in a patient with Philadelphia chromosome positive T-Cell acute lymphoblastic leukemia: a rare case report and review of literature. Case Rep Oncol. 2021;14(2):1040–50. 10.1159/000516270. 34326740 10.1159/000516270 PMC8299423

