# Supplementary material for: Molecular background of Philadelphia chromosome dependent enhancement of cellular growth and tyrosine kinase inhibitor sensitivity
Source: Exp Hematol Oncol. 2026 Feb 19;15:26. doi: 10.1186/s40164-026-00758-4 (PMC12922320; doi:10.1186/s40164-026-00758-4)
Supplement: Supplementary file 1 — Supplementary Material 1. [file 40164_2026_758_MOESM1_ESM.docx]

**Molecular background of Philadelphia Chromosome dependent enhancement of cellular growth and tyrosine kinase inhibitor sensitivity**

**Supplementary Figures and Figure Legends:**


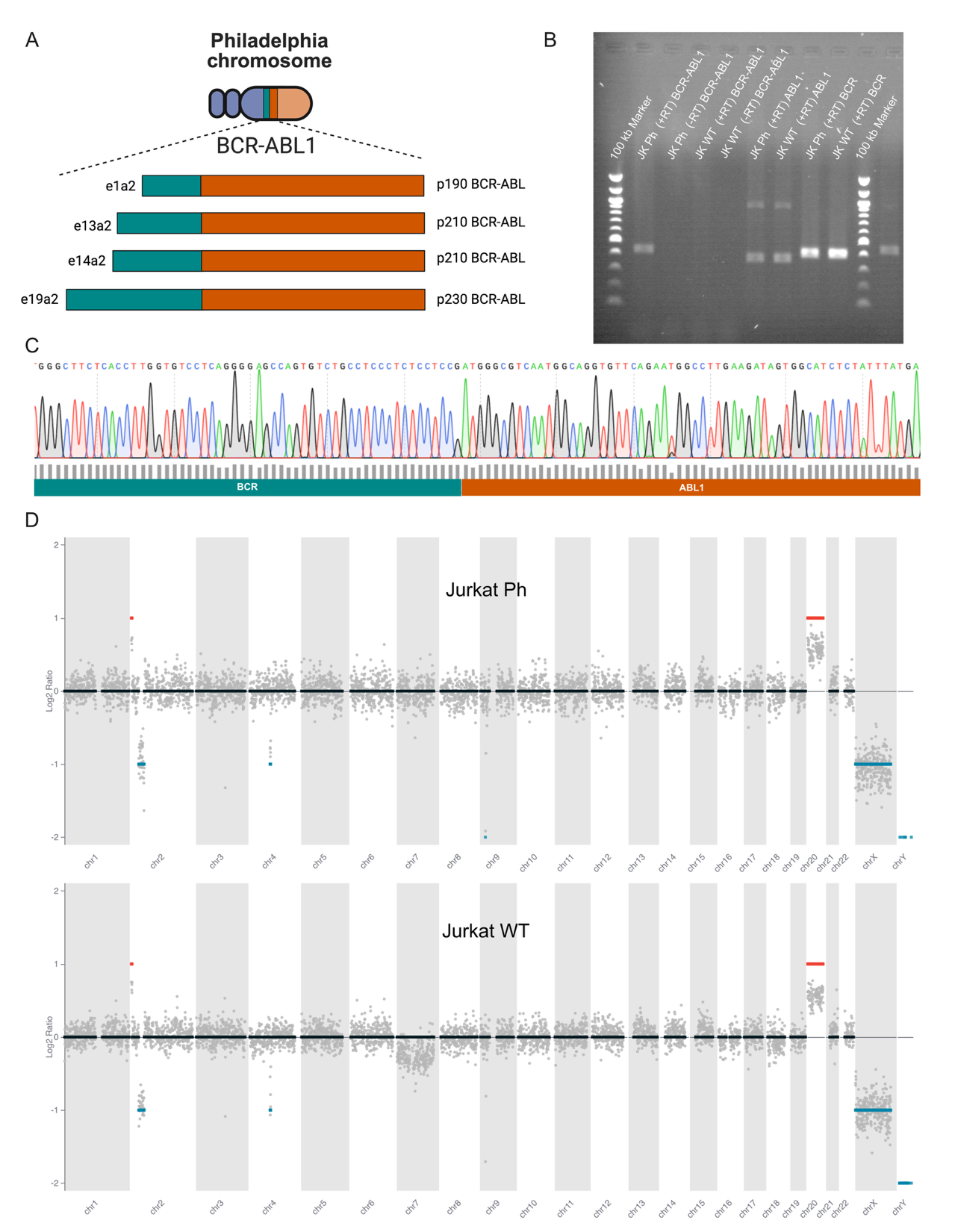


**Supplemental Figure S1.** Validation and molecular characterization of the Jurkat-Ph model. **A)** Schematic overview *and* examples of mRNA transcripts generated from different Philadelphia chromosome breakpoints. **B)** RT–PCR detection of BCR, ABL1, and BCR-ABL1 fusion transcripts. PCR was performed with (+RT) and without (*-*RT) reverse transcriptase to exclude genomic DNA contamination, and products were visualized by agarose gel electrophoresis. **C)** Confirmation of the BCR-ABL1 fusion by Sanger sequencing. Sections from the BCR gene (blue) and sections from the ABL1 gene (orange) are marked and highlighted, respectively. The continuous signal confirmed the fusion and homogeneity of the cells containing the BCR-ABL1 fusion transcript. **D)** Whole-genome copy-number profiles of Jurkat-WT and Jurkat-Ph cells generated using the Oxford Nanopore Technologies wf-cnv workflow. The Y-axis represents log2 copy-number ratios derived from normalized read depth relative to an inferred diploid baseline across all chromosomes (X-axis). Both cell lines show similar karyotypes across all chromosomes (X-axis) and comparable copy-number alterations (Y-axis), indicating that introduction of the BCR-ABL1 fusion did not generate major chromosomal abnormalities.


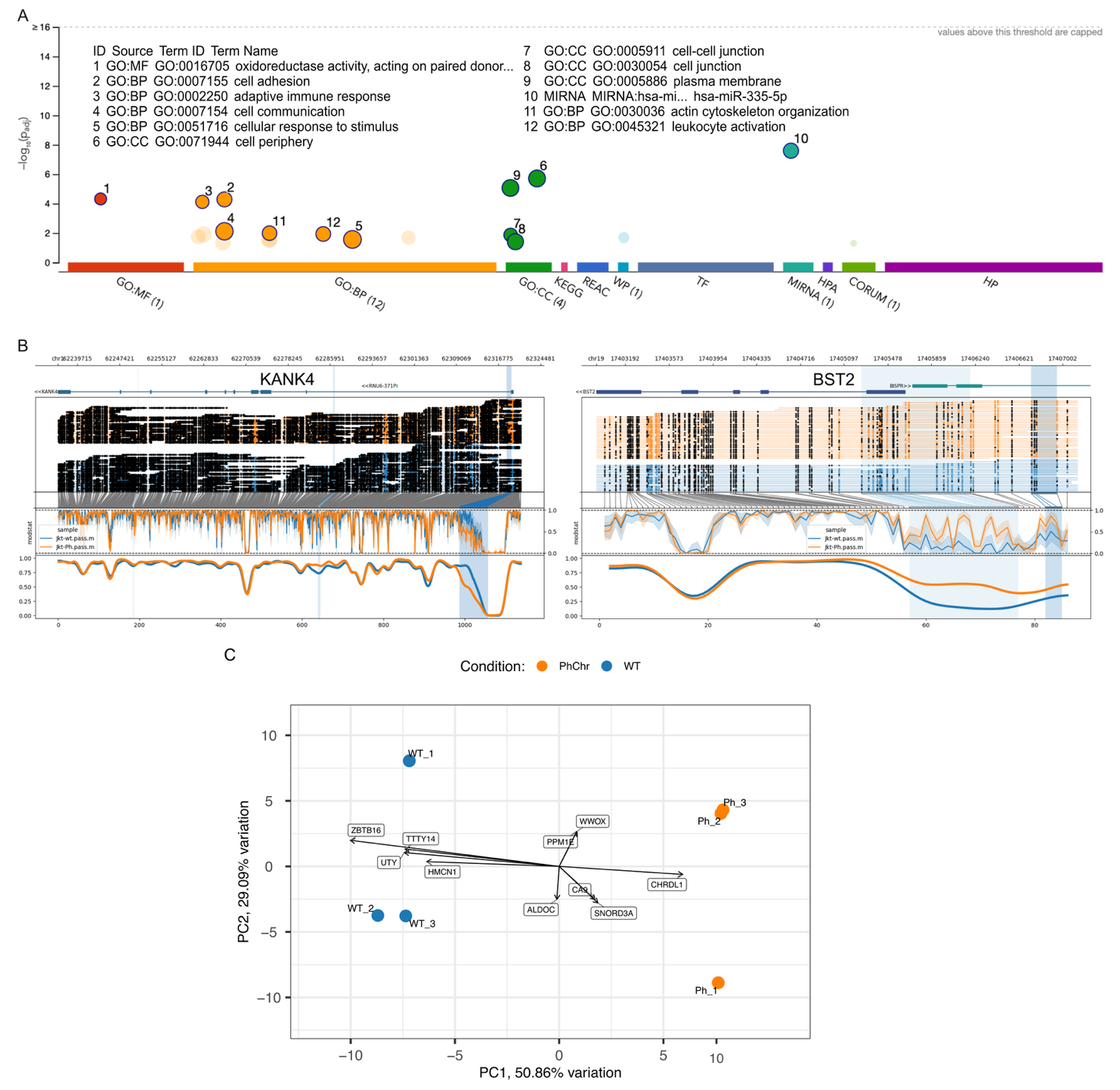


**Supplemental Figure S2.** **A**) g: Profiler enrichment analysis of downregulated proteins. GO term names were manually added to the figure based on the corresponding numerical identifiers. **B**) DNA-methylation profiles of genes displaying canonical promoter–expression relationships. Each gene panel shows promoter regions, gene bodies, and flanking sequences with methylation levels in Jurkat-WT (blue) and Jurkat-Ph (orange). Differentially methylated regions (DMRs) are highlighted. KANK4 and BST2 exemplify genes with promoter hypomethylation–upregulation and promoter hypermethylation–downregulation patterns, respectively. **C)** PCA plot of RNA-seq data showing clear separation between Jurkat-Ph and Jurkat-WT cells. Variance-stabilized (rlog-transformed) read counts were used for visualization.

## Supplemental materials and methods

### Cell culture

Jurkat cells were grown as a suspension culture in a T75 cell culture flask in RPMI 1640 medium (Thermo Fisher Scientific) supplemented with 1% penicillin/streptomycin (Gibco) and 10% fetal bovine serum (FBS). The suspension culture was maintained in a 37°C incubator with 5% CO_2_. The cells were passaged manually every 3 to 4 days.

### CRISPR/Cas9

CRISPR/Cas9 modification was performed via the Alt-R RNP system (Alt-R S.p. Cas9 Nuclease V3, IDT). Target-specific crRNAs were designed against the BCR and ABL1 breakpoint regions via CRISPOR and duplexed with the universal tracrRNA (IDT) to generate functional guide RNAs [1]. A list of crRNAs and corresponding primers is provided in supplementary material 2. For genome editing, Cas9 nuclease was complexed with duplex crRNA:tracrRNA to form ribonucleoprotein (RNP) complexes, which were delivered into Jurkat cells following the manufacturer’s protocol (IDT Alt-R RNP system). RNP transfection was performed following the manufacturer’s protocol with the Neon Transfection System (Thermo Fisher). The cells were washed 2x with PBS and counted via a TC20 Automated Cell Counter (Bio-Rad). A total of 50,000 cells were resuspended in buffer R. The cell suspension was mixed with RNP and an electroporation enhancer (IDT) and electroporated with 3 pulses for 10 ms at 1600 V. The cells were then transferred into 6-well plates, and after 72 hours, the medium was changed to recapitulate normal culture conditions. The cells were transferred to a T25 flask after 72 hours of growth in 6-well plates.

### Screening for transfected colonies

The cells were diluted to a very low concentration and transferred to a 96-well plate via serial dilution so that one cell grew per well in standard RPMI medium. Clonally selected cells were screened for mutations via PCR and Sanger sequencing. The sequencing data were analyzed with the TIDE [2] and Indigo [3] software tools. The 3 most likely off-target effects (determined by combining the CRISPOR and ccTOP results) were also amplified via PCR, sequenced and analyzed in the same fashion.

**Tyrosine kinase inhibitor treatment**

To assess the impact of tyrosine kinase inhibitors on cell proliferation, both Jurkat wild-type (WT) and BCR-ABL1-positive (Ph) cells were cultivated in the presence of either imatinib (5 µM) or dasatinib (50 nM). Imatinib mesylate (Cat. No. 72532) and dasatinib (Cat. No. 73082) were purchased from Stem Cell Technologies (Vancouver, BC, Canada) and dissolved in DMSO to prepare 10 mM stock solutions. A total of 50,000 viable cells were seeded per T75 flask for each condition. The cells were maintained in the presence of TKI for a period of 13 days, and the number of viable cells was measured at regular intervals (every second day, or as specified) via trypan blue exclusion. To evaluate the dose-dependent effects, Jurkat-WT and Jurkat-Ph cells were seeded at an initial density of 100,000 cells per T25 flask and treated with increasing concentrations of TKIs. To maintain cell viability during the extended treatment, partial medium renewal was performed on days 3 and 5 of culture. For this purpose, cells were centrifuged at 500 × g for 5 minutes, followed by the removal of 5 mL of culture medium and replacement with 5 mL of fresh standard RPMI-1640 medium. This procedure resulted in an approximately fourfold dilution (0.25) of the originally applied TKIs after day 5. On day 7, viable cells were counted via a TC20 Automated Cell Counter (Bio-Rad). The corresponding calculated concentrations after dilution were as follows:

| Compound | Initial concentration (Day 0) | Estimated concentration after dilution (Day 3) | Estimated concentration after dilution (Day 5) |
| --- | --- | --- | --- |
| Imatinib | 10 µM, 50 µM, 100 µM | 5 µM, 25 µM, 50 µM | 2.5 µM, 12.5 µM, 25 µM |
| Dasatinib | 10 nM, 50 nM, 100 nM | 5 nM, 25 nM, 50 nM | 2.5 nM, 12.5 nM, 25 nM |

The starting concentrations selected for this study were determined to represent clinically relevant ranges and to account for the differing potencies and pharmacodynamics of the two inhibitors. In vitro studies have demonstrated that, compared with imatinib, dasatinib has 325-fold greater potency in cells transduced with unmutated BCR-ABL1 [4]. All the experiments were performed in triplicate.

### DNA isolation/PCR

DNA was extracted by lysing the cells and digesting the proteins in buffer (5 mM EDTA, 200 mM NaCl, 100 mM Tris, 0.2% SDS, 0.2 mg/ml proteinase K) for 2 h at 55°C, followed by DNA precipitation with isopropanol. start PCR was performed to amplify the fusion region of interest via the primers 5’-ACGTGTGTTGTCAGGAGAGT-3’ and 5’-AGTGCCTGTGACTGTCTCTG-3’ and HS Taq Mastermix (Biozym) on a SureCycler 8800 thermal cycler (Agilent Technologies). The PCR conditions included initial denaturation at 95°C for 5 minutes, followed by 30 cycles of 95°C for 30 seconds, 58°C for 30 seconds, and 72°C for 1 minute, with a final extension at 72°C for 5 minutes. The amplified products were resolved on a 1% agarose gel to confirm the presence of the expected DNA fragment.

### RT‒PCR

The isolation of Jurkat cell mRNA was carried out following the manufacturer's protocol, utilizing a commercial kit (RNASolv, Omega Bio-Tek). The isolated RNA was subsequently subjected to DNAseI treatment (Thermo Fisher Scientific) as recommended by the manufacturer. The quantification of isolated RNA was conducted via a Qubit RNA assay kit (Thermo Fisher Scientific), and the quality assessment was performed via an Agilent 2100 Bioanalyzer. The RNA was reverse transcribed into cDNA via the SuperScript™ IV Reverse Transcriptase Kit (Thermo Fisher Scientific).

To detect the BCR-ABL1 p190 fusion transcript, PCR was performed via the following fusion-specific primers: forward (BCR exon 1): 5′- CTCGCAACAGTCCTTCGACA-3′. Reverse (ABL1 exon 2): 5′- AGACTGTTGACTGGCGTGAT-3′. The PCR conditions included initial denaturation at 95°C for 2 minutes, followed by 30 cycles of 95°C for 30 seconds, 58°C for 20 seconds, and 72°C for 20 seconds, with a final extension at 72°C for 3 minutes. The products were resolved on a 1% agarose gel. The expression of the fusion transcript was confirmed by the presence of a band of approximately 480 base pairs.

### Mycoplasma test

A test for mycoplasma contamination was performed via the MycoSPY Kit (Biontex) according to the manufacturer’s recommendations.

### Cell line authentication

To verify the identity and purity of the Jurkat cell lines used in this study, short tandem repeat (STR) profiling was performed. Cell pellets from both Jurkat-WT and Jurkat-Ph cells were sent to Eurofins Genomics for authentication. STR profiling confirmed the identity of both cell lines with no signs of cross-contamination.

### Evaluation of chromosomal integrity

Genomic DNA was extracted from Jurkat-Ph and Jurkat wild-type cells, quantified, and assessed for quality using a 4150 TapeStation System (Agilent). Chromosomal copy number profiles were generated from Oxford Nanopore sequencing data using the wf-cnv Nextflow workflow provided by Oxford Nanopore Technologies. Briefly, sequencing reads were aligned to the human reference genome (GRCh38), and genome-wide copy number changes were inferred based on read depth. Log₂ copy number ratios were calculated and visualized to assess chromosomal integrity and to compare large-scale copy number alterations between Jurkat-Ph and Jurkat-WT cells.

### RNA sequencing (RNA-seq) and data analysis

Jurkat cell total RNA was isolated via RNASolv (Omega Bio-Tek) according to the manufacturer’s protocol and treated with DNAse I (Thermo Fisher). The isolated RNA was quantified (Qubit RNA assay kit, Thermo Fisher Scientific), and its quality was assessed via a 2100 Bioanalyzer System (Agilent). Samples with a high RNA integrity number (RIN > 8) were selected for library preparation. RNA-seq libraries were prepared from 100 ng of rRNA-depleted RNA via the NEBNext Ultra II Directional RNA Library Kit (NEB) following the manufacturer’s protocol. All libraries were paired-end sequenced on an Illumina NextSeq550 platform at a depth of 23 million reads each (2x75 bp) on a midoutput flowcell.

The read quality of the RNA-seq data was assessed via FastQC (v0.11.4). The raw sequence reads were mapped against the human reference genome (genome build GRCh38) via the STAR splice-aware aligner (v. 2.7.11b) with default settings [5]. The fraction of reads that mapped uniquely was, on average, 88%. To count the reads matching genes, we used HTSeq-count (v. 0.11.3) [6], which has an approximately 80% assignment success rate. Differential expression analysis was performed by comparing Jurkat-Ph samples to wild-type controls via DESeq2 [7].

Differentially expressed genes (DEGs) identified by DESeq2 were subjected to functional enrichment analysis. Over representation analysis was performed using the clusterProfiler R package [8]. Gene set enrichment analysis was performed using g:Profiler with default settings, applying multiple testing correction using the Benjamini–Hochberg method (FDR < 0.05). Enrichment analyses were conducted using a user-defined background gene list consisting of all genes with at least two mapped reads. Gene Ontology (GO) gene set collections were used to identify enriched biological processes, and MSigDB Hallmark gene sets were analyzed to assess coordinated transcriptional programs. Only terms with adjusted *p* values below 0.05 were considered significantly enriched.

For PCA plots, read counts were transformed using the rlog function of DESeq2, which stabilizes variance across the dynamic range of expression. This transformation does not influence the outcome of the differential expression analysis, which is based on raw count data normalized within DESeq2

### DNA methylation analysis

For global methylome analysis, the DNA of Jurkat-WT and Jurkat-Ph cells was isolated according to standard procedures via the Maxwell® RSC Blood DNA Kit (Promega). A total of 250 ng of genomic DNA was subjected to bisulfite conversion via the EZ DNA Methylation-Lightning Kit (Zymo Research) according to the manufacturer’s protocol. Illumina ® Infinium MethylationEPIC v2.0 BeadChip array analysis was performed on representative samples from each condition to obtain genome-wide methylation profiles covering approximately 935,000 CpG sites. Raw data from Illumina Epic v2.0 BeadChip arrays were preprocessed, and beta values, such as methylation level estimation, were extracted via the R package Sesame (v.1.22.2). CpG sites with missing values were excluded. Differential methylation was quantified as differences in beta values (Δβ) between Jurkat-Ph and Jurkat-WT cells. To expand coverage beyond predefined CpG sites, both Jurkat-WT and Jurkat-Ph cell lines were also analyzed via Oxford Nanopore sequencing. A high-molecular-weight DNA library was prepared via the LSK114 library preparation kit (Oxford nanopore) and sequenced on the P2 Solo platform. Basecalling was performed with Dorado (model sup v4.2.0, methylation-aware model v4.2.0), and the reads were aligned to the human reference genome (hg38). This sequencing-based approach enables the detection of methylation patterns across broader genomic regions, including promoters and gene bodies, providing additional insights into methylation changes not captured by array-based methods.

Differentially methylated regions (DMRs), defined as genomic regions with consistent methylation differences across multiple CpG sites, were identified between Jurkat-WT and Jurkat-Ph cells using the R package DSS [9]. Global and locus-specific methylation patterns were visualized with the methylartist toolkit [10]. To investigate potential regulatory effects, DMRs were further analyzed for overlapping transcription factor-binding sites via JASPAR tracks within the UCSC Genome Browser [11].

### Sample preparation for mass spectrometry

The cell pellets were lysed in 75μl of lysis buffer (4% sodium deoxycholate, 100 mM Tris-HCl pH 8.5) and incubated at 95°C for 7 minutes. The DNA was sheared via sonication (Bandelin BR30; 4x 30 s, cycle number 0.5, amplitude 100%). Lysates were cleared by centrifugation for 20 minutes at 17,000 xg. The protein concentration was determined via the microBCA assay (Thermo Fisher). Lysates containing 4 µg and 20 µg of total protein were used for proteome and phosphoproteome analyses, respectively. The reduction and alkylation of disulfide bonds on cysteine residues were performed with tris(2-carboxyethyl)phosphine and 2-chloroacetamide at final concentrations of 10 mM and 40 mM, respectively, for 10 min at 45°C. Proteome samples were further processed via a bead-based SP3 protocol [12]. Proteins were digested via a trypsin/Lys-C mixture at a protease:protein ratio of 1:25 at 37°C for 16 h. Digestion of global proteome samples was stopped by the addition of TFA. The magnetic beads were removed, and the supernatants were transferred to glass vials and stored at -20°C until MS analysis. Following SP3 based digestion, phosphopeptides were enriched following the µPhos protocol [13]. Half of the generated phosphopeptide sample was loaded onto Evotips and stored at 4°C until measurement.

### Mass spectrometry and data analysis

Global proteome analysis was conducted on an Ultimate 3000 nano-liquid chromatography system coupled online to a Q-Exactive HF mass spectrometer (Thermo Fisher Scientific, Germany). Mass spectra were acquired in the Data Independent Acquisition (DIA) mode. The recorded MS data were analyzed using the DirectDIA+ mode in Spectronaut (Version 17.6.230428.55965, Biognosys, Switzerland), utilizing the UniProt *Homo sapiens* reference proteome (downloaded January 2022). Carbamidomethylation on cysteine residues was set as a fixed modification, while oxidation on methionine and protein N-terminal acetylation was defined as variable modifications. Further processing of the generated data and statistical analysis was conducted in R (Version 4.4.1) using the ROTS test implemented in the SpectroPipeR package (Version 0.4.0) [14].

Phosphoproteome samples were analyzed using an Evosep One system coupled to a timsTOF Pro 2 mass spectrometer (Bruker Daltonics, Germany). Mass spectra were acquired in DIA-PASEF mode, leveraging the method previously described The recorded MS data were analyzed using the DirectDIA+ mode in Spectronaut (Version 17.6.230428.55965, Biognosys, Switzerland) using the Uniprot *homo sapiens* reference proteome (downloaded 01/2022). Carbamidomethylation on cysteine was set as a fixed modification. Variable modifications included phosphorylation on serine, threonine, and tyrosine residues, oxidation on methionine residues. Initial data processing was performed in Perseus (Version 2.0.6) [15] using the peptide collapse plug in [16] and subsequently in R (Version 4.4.1). Only phosphosites with a localization probability > 0.75 were included and considered for statistical analysis. Sites not present in at least three samples of one group were removed. Missing values were imputed from the normal distribution of each sample, using a 1.8 standard deviation down shift and a width of 0.3. Samples were normalized by median subtraction. Differential protein and phosphosite abundance were assessed using a Welch's T-test, followed by False Discovery Rate (FDR) calculation using the qvalue package [17]. Proteins or phosphosite with FDR < 0.05 were considered significant.

## References

[1] J.-P. Concordet and M. Haeussler, “CRISPOR: intuitive guide selection for CRISPR/Cas9 genome editing experiments and screens,” *Nucleic Acids Research*, vol. 46, no. W1, pp. W242–W245, July 2018, doi: 10.1093/nar/gky354.

[2] E. K. Brinkman, T. Chen, M. Amendola, and B. van Steensel, “Easy quantitative assessment of genome editing by sequence trace decomposition,” *Nucleic Acids Research*, vol. 42, no. 22, p. e168, Dec. 2014, doi: 10.1093/nar/gku936.

[3] T. Rausch, M. H.-Y. Fritz, A. Untergasser, and V. Benes, “Tracy: basecalling, alignment, assembly and deconvolution of sanger chromatogram trace files,” *BMC Genomics*, vol. 21, no. 1, p. 230, Mar. 2020, doi: 10.1186/s12864-020-6635-8.

[4] T. O’Hare *et al.*, “In vitro Activity of Bcr-Abl Inhibitors AMN107 and BMS-354825 against Clinically Relevant Imatinib-Resistant Abl Kinase Domain Mutants,” *Cancer Research*, vol. 65, no. 11, pp. 4500–4505, June 2005, doi: 10.1158/0008-5472.CAN-05-0259.

[5] A. Dobin *et al.*, “STAR: ultrafast universal RNA-seq aligner,” *Bioinformatics*, vol. 29, no. 1, pp. 15–21, Jan. 2013, doi: 10.1093/bioinformatics/bts635.

[6] S. Anders, P. T. Pyl, and W. Huber, “HTSeq—a Python framework to work with high-throughput sequencing data,” *Bioinformatics*, vol. 31, no. 2, pp. 166–169, Jan. 2015, doi: 10.1093/bioinformatics/btu638.

[7] M. I. Love, W. Huber, and S. Anders, “Moderated estimation of fold change and dispersion for RNA-seq data with DESeq2,” *Genome Biology*, vol. 15, no. 12, p. 550, Dec. 2014, doi: 10.1186/s13059-014-0550-8.

[8] S. Xu *et al.*, “Using clusterProfiler to characterize multiomics data,” *Nat Protoc*, vol. 19, no. 11, pp. 3292–3320, Nov. 2024, doi: 10.1038/s41596-024-01020-z.

[9] H. Feng, K. N. Conneely, and H. Wu, “A Bayesian hierarchical model to detect differentially methylated loci from single nucleotide resolution sequencing data,” *Nucleic Acids Research*, vol. 42, no. 8, p. e69, Apr. 2014, doi: 10.1093/nar/gku154.

[10] S. W. Cheetham, M. Kindlova, and A. D. Ewing, “Methylartist: tools for visualizing modified bases from nanopore sequence data,” *Bioinformatics*, vol. 38, no. 11, pp. 3109–3112, May 2022, doi: 10.1093/bioinformatics/btac292.

[11] I. Rauluseviciute *et al.*, “JASPAR 2024: 20th anniversary of the open-access database of transcription factor binding profiles,” *Nucleic Acids Research*, vol. 52, no. D1, pp. D174–D182, Jan. 2024, doi: 10.1093/nar/gkad1059.

[12] C. S. Hughes, S. Moggridge, T. Müller, P. H. Sorensen, G. B. Morin, and J. Krijgsveld, “Single-pot, solid-phase-enhanced sample preparation for proteomics experiments,” *Nat Protoc*, vol. 14, no. 1, pp. 68–85, Jan. 2019, doi: 10.1038/s41596-018-0082-x.

[13] D. Oliinyk *et al.*, “µPhos: a scalable and sensitive platform for high-dimensional phosphoproteomics,” *Molecular Systems Biology*, vol. 20, no. 8, pp. 972–995, Aug. 2024, doi: 10.1038/s44320-024-00050-9.

[14] S. Michalik *et al.*, “SpectroPipeR—a streamlining post Spectronaut® DIA-MS data analysis R package,” *Bioinformatics*, vol. 41, no. 3, p. btaf086, Mar. 2025, doi: 10.1093/bioinformatics/btaf086.

[15] S. Tyanova *et al.*, “The Perseus computational platform for comprehensive analysis of (prote)omics data,” *Nat Methods*, vol. 13, no. 9, pp. 731–740, Sept. 2016, doi: 10.1038/nmeth.3901.

[16] D. B. Bekker-Jensen *et al.*, “Rapid and site-specific deep phosphoproteome profiling by data-independent acquisition without the need for spectral libraries,” *Nat Commun*, vol. 11, no. 1, p. 787, Feb. 2020, doi: 10.1038/s41467-020-14609-1.

[17] J. D. Storey, “A direct approach to false discovery rates,” *Journal of the Royal Statistical Society: Series B (Statistical Methodology)*, vol. 64, no. 3, pp. 479–498, 2002, doi: 10.1111/1467-9868.00346.
